# State of the psychometric methods: patient-reported outcome measure development and refinement using item response theory

**DOI:** 10.1186/s41687-019-0130-5

**Published:** 2019-07-30

**Authors:** Angela M. Stover, Lori D. McLeod, Michelle M. Langer, Wen-Hung Chen, Bryce B. Reeve

**Affiliations:** 1 Department of Health Policy and Management, University of North Carolina at Chapel Hill, 1101-G McGavran-Greenberg Hall (CB# 7411), Chapel Hill, NC 27599 USA; 2 Lineberger Comprehensive Cancer Center, University of North Carolina at Chapel Hill School of Medicine, 101 Manning Drive, Chapel Hill, NC 27599 USA; 3 RTI Health Solutions, 3040 Cornwallis Road, Research Triangle Park, NC 27709-2194 USA; 4 Current affiliation: Medical Social Sciences; Feinberg School of Medicine, Northwestern University, 625 N Michigan Ave Suite 2700, Chicago, IL 60611 USA; 5 Current affiliation: Center for Health Measurement Department of Population Health Sciences and Pediatrics, Duke University School of Medicine, 2200 West Main St, Suite 720A, Durham, NC 27707 USA

**Keywords:** Item response theory, Scale construction, Scale evaluation, Measurement, PROMIS®

## Abstract

**Background:**

This paper is part of a series comparing different psychometric approaches to evaluate patient-reported outcome (PRO) measures using the same items and dataset. We provide an overview and example application to demonstrate 1) using item response theory (IRT) to identify poor and well performing items; 2) testing if items perform differently based on demographic characteristics (differential item functioning, DIF); and 3) balancing IRT and content validity considerations to select items for short forms.

**Methods:**

Model fit, local dependence, and DIF were examined for 51 items initially considered for the Patient-Reported Outcomes Measurement Information System® (PROMIS®) Depression item bank. Samejima’s graded response model was used to examine how well each item measured severity levels of depression and how well it distinguished between individuals with high and low levels of depression. Two short forms were constructed based on psychometric properties and consensus discussions with instrument developers, including psychometricians and content experts. Calibrations presented here are for didactic purposes and are not intended to replace official PROMIS parameters or to be used for research.

**Results:**

Of the 51 depression items, 14 exhibited local dependence, 3 exhibited DIF for gender, and 9 exhibited misfit, and these items were removed from consideration for short forms. Short form 1 prioritized content, and thus items were chosen to meet DSM-V criteria rather than being discarded for lower discrimination parameters. Short form 2 prioritized well performing items, and thus fewer DSM-V criteria were satisfied. Short forms 1–2 performed similarly for model fit statistics, but short form 2 provided greater item precision.

**Conclusions:**

IRT is a family of flexible models providing item- and scale-level information, making it a powerful tool for scale construction and refinement. Strengths of IRT models include placing respondents and items on the same metric, testing DIF across demographic or clinical subgroups, and facilitating creation of targeted short forms. Limitations include large sample sizes to obtain stable item parameters, and necessary familiarity with measurement methods to interpret results. Combining psychometric data with stakeholder input (including people with lived experiences of the health condition and clinicians) is highly recommended for scale development and evaluation.

## Background

Patient-reported outcome (PRO) measures quantify the impact of health conditions and treatments on people’s lives with respect to how they feel, function, and perceive their health-related quality of life [1]. PROs are routinely used to inform  clinical trial endpoints (see [2–4]) and are increasingly used in drug labeling claims [1, 5, 6]. In routine care, systematically administered PROs improve detection of symptoms, and enhance clinician-patient communication and patients’ satisfaction with care [7–10]. Given this variety of uses, robust PRO measure development and evaluation is critical. Initiatives such as the Patient-Reported Outcomes Measurement Information System® (PROMIS®) [11, 12] and the European Organization for Research and Treatment of Cancer (EORTC) [13, 14] are examples of international programs leading PRO measure development.

Psychometric theory and its statistical models play an important role in developing and evaluating PRO measures. For example, there is a large literature on applying statistical methods such as item response theory (IRT) to develop and evaluate PRO measures (e.g., [15–20]). However, little PRO literature is available directly comparing different psychometric approaches and content considerations. This paper is part of a series comparing psychometric approaches (IRT, classical test theory, and Rasch analysis) using the same items and dataset [21].

The objective of the current paper is to provide an overview and example application demonstrating how IRT and content validity considerations can be used to develop and refine PRO measures. Description of an example software application (IRTPRO) and sample output are provided [22]. IRT methodology is applied to 51 items considered for the initial PROMIS Depression item bank [23] that were tested as part of wave 1 testing [11, 12]. The official PROMIS® Depression item bank has a subset of 28 items because items with psychometric or content issues were eliminated [11, 12, 23, 24]. The larger Depression item bank of 51 items was chosen for the current paper so there would be more opportunities to identify problematic psychometric issues. We demonstrate: 1) using IRT to identify poor and well performing items; 2) testing if items perform differently based on demographic characteristics (using differential item functioning [DIF] testing); and 3) balancing IRT and content validity considerations to select items for short forms.

The PROMIS® system includes several versions of Depression short forms including a subset of items from the 28-item calibrated bank [23]. For this paper, we purposely created new short forms based at least in part on the Diagnostic and Statistical Manual version 5 (DSM-V) [25] to avoid duplication with prior work. The calibrations and short forms presented in this series are for didactic purposes and are not intended to be used for research or to replace official PROMIS® versions (see www.healthmeasures.net).

### IRT overview

IRT is a family of statistical models providing information about the performance of items and the scales they comprise through the analysis of item responses. IRT has been widely used in the education field for over 40 years and is increasingly used to develop and evaluate PRO measures. Unidimensional (single factor) and multi-dimensional (more than one factor) IRT models are available but this paper focuses on unidimensional IRT models. Readers interested in multidimensional IRT are referred to these sources [26, 27].

IRT-calibrated item banks (such as PROMIS) offer multiple options for instrument development and refinement, including creating customized short forms comprised of a fixed set of items or administering a tailored assessment through computerized adaptive testing (CAT). Because items are linked (or calibrated) by the IRT model, scores can be compared across administrations or different samples using IRT scaling. IRT-derived scores typically are more sensitive in cross-sectional tests and are more responsive to changes in health over time than scores for the same item set produced using classical methods [19].

IRT models are mathematical equations that describe the relationship between the latent trait being measured (commonly denoted as theta: *θ*), such as depression level, and the probability of endorsing a given response to an item that serves as an indicator of the trait being measured. A popular IRT model used in PRO research is Samejima’s [28, 29] graded response model (GRM), which can be used when there are three or more response choices for each item within the PRO measure. In the current example, the PROMIS® Depression items have five response options (never, rarely, sometimes, often, always) to measure frequency of depression symptoms.

**Fig. 1:**
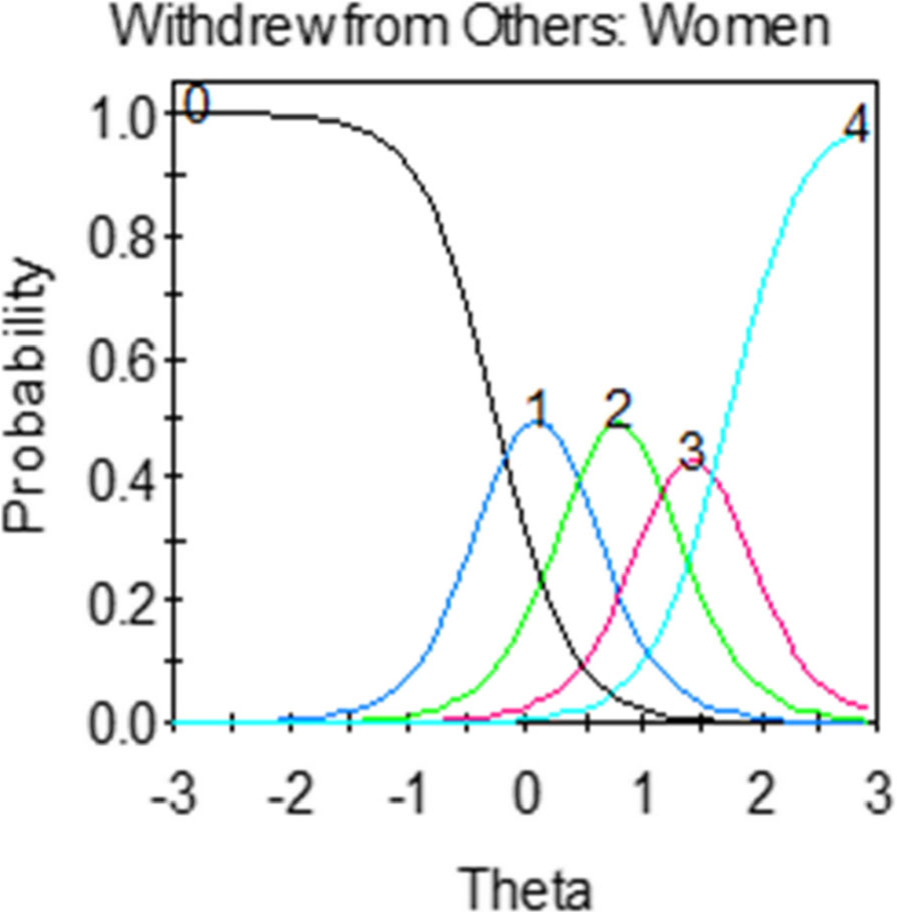
Example of an item characteristic curve (ICC) or “trace line”. Vertical axis: probability of endorsement for each response category; Horizontal axis: theta (level of latent trait, e.g., level of depressive symptoms). Response option choices: 0: Never; 1: Rarely; 2: Sometimes; 3: Often; 4: Always

A helpful feature of IRT is the ability to produce visual images of how well items are performing. Item characteristic curves (ICCs) or “trace lines” [30] visually depict the relationship between the probability of item responses and *θ*. In graphical form, ICCs display arcs or curves for each response option on the same graph. Figure 1 is an example of ICCs for an item with five response options. The vertical axis of the ICC graph represents the probability of endorsement and the horizontal axis is *θ* (depression level). By convention, the *θ* metric is standardized to a mean of 0 and standard deviation of 1, but can be converted to any metric using a linear transformation. ICCs are valuable for determining the appropriate number of response categories based on patients’ responses and the latent trait level that an individual likely has in order to endorse each response option. Additionally, both items and individuals can be characterized on a common metric along the latent trait continuum.

Graphical representations of ICCs are derived from a formula. In the GRM, the formula for ICCs (trace lines) is:

$$ P\left({x}_i=\left.j\right|\theta \right)=\frac{1}{1+\mathit{\exp}\left[-{a}_i\left(\theta -{b}_{ij}\right)\right]}-\frac{1}{1+\mathit{\exp}\left[-{a}_i\left(\theta -{b}_{ij+1}\right)\right]}, $$

which states that the probability of responding in category *j* (e.g., response option of “rarely”) is the difference between trace lines for the probability of responding in category *j* or higher and category *j* + 1 or higher. In the formula, *a*_*i*_ is the item slope (or discrimination) parameter and *b*_*ij*_ are the item threshold (or severity) parameters [31]. The slope parameter (*a*) measures the strength of the relationship between the item and *θ.*

Values of *a* range from 0 to infinity, where higher slopes indicate that the item discriminates more sharply between individuals above and below a particular value of *b*. For example, depression items with larger slopes are better able to distinguish between individuals with higher and lower levels of depression.

Threshold parameters (*b*_*ij*_) represent the points along the latent trait at which a respondent whose *θ* = *b*_*ij*_ has a 0.5 probability of selecting response option *j* or higher. The higher the *b*-parameters, the higher the trait level a respondent needs to have in order to endorse that response option.

The GRM produces C – 1 estimated *b*-parameters, where C is the number of response options. For an item with five response categories (common in PROs), there are four thresholds (*b*_*1*_*-b*_*4*_). *b*_*1*_ is the threshold for the trace line describing the probability of selecting response options 1,  2, 3, or  4; *b*_*2*_ is for response options 2,  3, or  4; *b*_*3*_ is for response options 3 or 4; and *b*_*4*_ is for response option 4. Values of *b*-parameters typically range from − 3 to + 3 and are interpreted as standard deviations showing the range of *θ* (depression level) covered by the item. For example, if *b*_*1*_ is 0 and *b*_*4*_ is 3, the item provides the most information about depression from the mean to three standard deviations above the mean. Table 1 provides an overview of key concepts used in IRT. Readers interested in the statistical underpinnings of IRT are referred to [17, 32].

**Table 1:** Common terms used in an IRT graded response model

Term	Abbreviation/Symbol	Description
Slope parameter	*a*	• Also referred to as the discrimination parameter. • Measures the strength of the relationship between the item and the latent variable being measured. • Items with larger slopes are better able to distinguish between individuals with higher and lower levels of the latent variable being measured.
Threshold parameters	*b*_*ij*_	• Also known as the location parameters or the difficulty/severity parameters. • Represents the points along theta at which the corresponding response categories are the most discriminating or informative. • Items with higher thresholds represent greater severity of the latent variable being measured.
Theta	*Θ*	• Latent variable being measured (e.g., depression).
Item characteristic curve	ICC	• Also referred to as a “trace line.” • Visual image showing the probability of an item response across the range of theta (latent trait). • Can reveal weak items and overlapping response categories.
Test characteristic curve	TCC	• Sum of the ICCs across all items. • Shows the expected total summed score on the scale for each level of theta.
Item information function	IIF	• Index of the precision in measurement in distinguishing between individuals with different levels of the latent variable being measured. • More information indicates greater precision and reliability. • Item information is peaked when the slope parameter is high. • Standard error of measurement is inversely related to information.
Test information function	TIF	• Sum of the item information functions across all items. • Indicates where along theta the scale has the greatest measurement precision.
Item fit	*S-X*^*2*^	• Diagnostic statistic that examines goodness of fit of the IRT model for each item. • Examines observed and expected response proportions for each item value. • Significant result indicates item misfit.
Local dependence	LD	• Statistic that examines bivariate fit to identify evidence of items that are excessively related given the common underlying construct. • Significant result indicates content redundancy between two or more items.
Differential item functioning	DIF	• Measurement bias in an item between two or more groups while holding the latent trait level constant.

### Unidimensional IRT model assumptions

Unidimensional IRT models have four major assumptions: one dominant factor exists across the measure; items are locally independent; monotonicity; and model-data fit [15–20]. Unidimensionality indicates that the set of items assesses a single underlying trait. One paper in this series [33] conducted extensive analyses confirming that the PROMIS® depression items are unidimensional.

Local independence indicates each item is contributing uniquely to the latent trait being measured. Two items are said to be locally dependent if they contain extra association (co-variance) beyond what is measured by the latent trait. Model-data fit includes overall fit and individual item is fit for the specified IRT model [32]. Related to model-data fit, monotonicity is also assumed, indicating that the probability of endorsing response options indicative of higher levels of the measured trait (e.g., depression) increases with an individual’s level of the latent trait.

When a set of items satisfies the assumptions of one dominant factor, local independence, monotonicity, and model-data fit, the latent trait estimates are independent of the specific item set, and the item parameters are independent of the sample; these properties are a major advantage of IRT.

## Methods

### Item pool

The US National Institutes of Health (NIH)-funded PROMIS initiative developed item banks assessing common symptoms using state-of-the-art scale development methods [23, 34–36]. For the PROMIS Depression item bank used in this didactic example, an initial item pool consisting of over 500 items from more than 100 depression measures (proprietary measures were removed) were reviewed by clinical and measurement experts [23]. Focus groups were conducted with patients to enrich the item pool [35]. Items were categorized according to a priori depression subdomains of affect, cognition, behavioral (e.g., social withdrawal), somatic (e.g., sleep, weight), and suicidal ideation. Items judged to best represent each category were rewritten into a common format with a 7-day recall period. Three rounds of cognitive interviews were conducted with patients to ensure comprehensibility [36] before field testing the item bank. One item uses an intensity response scale (I lost weight without trying: “not at all,” “a little,” “somewhat,” “quite a bit,” “very much”), and the remaining 50 items use a frequency response scale (“never,” “rarely,” “sometimes,” “often,” “always”).

The current analyses use data for 51 items initially considered for inclusion in the PROMIS Depression item bank (28 items appear in the official calibrated bank) [23]. We used the larger item set as a didactic exercise to identify psychometric issues using IRT. The calibrations and short forms presented in this paper are not intended to replace official PROMIS parameters or to be used for research purposes. For instance, the parameters presented in the current paper have not been converted to a T-score metric centered on a census-matched sample like the PROMIS® Depression item bank. Additionally, we also used a different content criterion (DSM-V) than the original paper (affective and cognitive dimensions of depression [23]). For the current paper, IRB exemption was granted from the University of North Carolina at Chapel Hill.

### Overview of IRTPRO software

**Fig. 2:**
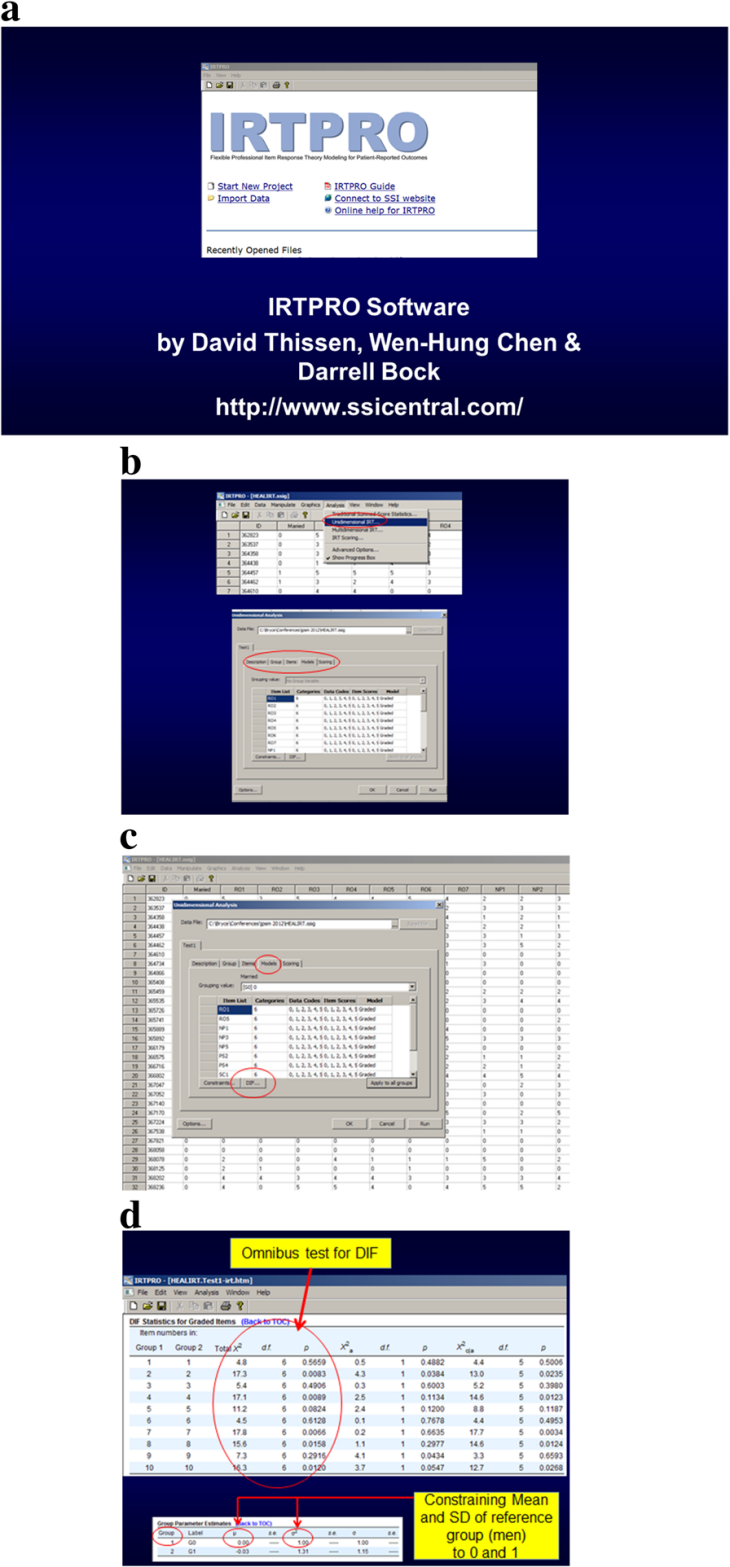
IRTPRO screen shots for graded response model and differential item functioning. **a** introductory screen. **b** first part of sequence to enable DIF detection **c** second part of sequence to enable DIF **d** DIF Output

IRTPRO software [22] was used to examine model fit, local independence, and DIF (see Fig. 2 for screen shots on how to run and interpret DIF). The user-interface is Windows-based, meaning that users “point and click” to invoke default settings for models but also have the flexibility to manually impose constraints on any parameter. IRTPRO offers a range of model choices, including: unidimensional and multidimensional models; one-, two-, and three-parameter logistic models [31, 37]; Samejima’s graded response model [28, 29]; generalized partial credit model [38, 39]; and the nominal response model [40, 41].

The default setting in IRTPRO for item parameter estimation/calibration is maximum likelihood [22] and we used the default. The default setting can be changed by specifying prior distributions for item parameters (in which case maximum a posteriori estimates would be computed) [22].

### Short-form development methods

A goal of this paper is to demonstrate the design of unidimensional short forms using a combination of IRT and content validity considerations. Content validity is the extent to which an item bank or short form captures key aspects of the concept it is supposed to measure. Two potential short forms were generated by consensus discussions conducted with psychometricians and content experts. Short form 1 prioritized items assessing DSM-V criteria for major depression: 1) depressed mood most of the day, every day, or markedly diminished interest or pleasure in activities; 2) at least five types of symptoms (significant weight gain or loss/appetite loss, insomnia/hypersomnia, psychomotor agitation or retardation, fatigue, worthlessness/excessive guilt, diminished ability to think/concentrate or indecisiveness, or suicidal ideation); and 3) clinically significant distress or impairment in social, occupational, or other areas [25]. Short form 2 prioritized well performing items in Table 2 (based on *a*- and *b*-parameters).

**Table 2:** Item parameters, fit statistics, local dependence, and DIF results for the 51 PROMIS® depression items

#	Item Stem	Item Parameters					Item Fit			*b-*DIF	
		*a*	*b*_*1*_	*b*_*2*_	*b*_*3*_	*b*_*4*_	*S-Χ*^*2*^	*d.f.*	LD	*Χ*^*2*^	*d.f.*
1	I felt hopeless	4.46	0.38	0.97	1.53	2.23	116.2	88			
2	I felt worthless	4.17	0.44	1.00	1.57	2.14	102.0	91			
3	I felt depressed	3.84	−0.11	0.59	1.29	2.15	*163.4*	120			
4	I felt unhappy	3.81	−0.39	0.41	1.27	2.06	151.5	120			
5	I felt that nothing could cheer me up	3.73	0.32	0.94	1.73	2.34	99.4	98			
6	I felt like a failure	3.73	0.19	0.72	1.58	2.05	137.2	116			
7	I felt helpless	3.65	0.35	0.94	1.67	2.36	115.0	105			
8	I felt that I wanted to give up on everything	3.6	0.56	1.1	1.6	2.3	111.3	95			
9	I felt that I had nothing to look forward to	3.3	0.31	0.8	1.5	2.3	147.1	117			
10	I felt that my life was empty	3.24	0.28	0.73	1.56	2.11	148.5	120			
11	I felt emotionally exhausted	3.32	−0.18	0.49	1.27	2.05	126.0	133			
12	I felt sad	3.20	−0.47	0.37	1.31	2.29	*205.7*	131			
13	I felt I had no reason for living	3.1	0.92	1.5	1.9	2.6	83.2	71	21,29,35		
14	I found that things in my life were overwhelming	3.1	−0.09	0.6	1.5	2.3	153.8	138			
15	I felt that I was not needed	3.08	0.21	0.84	1.55	2.38	162.4	127			
16	I felt disappointed in myself	3.05	−0.35	0.39	1.31	2.09	*199.9*	146			
17	I felt like I needed help for my depression	3.0	0.54	1.01	1.7	2.2	*150.2*	105			
18	I had trouble enjoying the things I used to enjoy	2.9	−0.09	0.60	1.4	2.2	165.2	146			
19	I felt discouraged about the future	2.92	−0.26	0.37	1.31	2.09	159.4	153			
20	I felt that I was to blame for things	2.88	−0.02	0.73	1.63	2.38	181.3	141			
21	I wished I were dead and away from it all	2.8	1.01	1.5	2.1	2.6	*103.3*	71	13,29,35		
22	I felt upset for no reason	2.83	0.23	0.93	1.85	2.93	135.3	117			
23	I felt that nothing was interesting	2.83	0.06	0.87	1.90	2.64	122.1	122			
24	I felt I was not as good as other people	2.8	0.15	0.80	1.6	2.3	161.5	130			
25	I withdrew from other people	2.72	0.04	0.70	1.47	2.30	160.5	144			
26	I had trouble making decisions	2.71	−0.16	0.75	1.66	2.57	174.8	140	32,38		
27	I had trouble feeling close to people	2.66	−0.12	0.55	1.41	2.24	153.6	155			
28	I felt pessimistic	2.65	−0.30	0.48	1.38	2.23	198.8	155			
29	I felt that others would be better off if I were dead	2.6	1.10	1.55	2.4	3.0	70.1	67	13,21,35		
30	I felt lonely	2.54	−0.09	0.60	1.45	2.21	186.0	158			
31	I felt unloved	2.51	0.26	0.90	1.76	2.48	161.8	136			
32	I had trouble thinking clearly	2.51	−0.15	0.78	1.84	2.84	178.7	145	26,38		
33	I had mood swings	2.45	−0.32	0.56	1.42	2.32	181.8	153			
34	I felt like crying	2.44	0.05	0.82	1.67	2.62	178.5	148	45	*43.4*	4
35	I thought about suicide	2.43	1.34	1.80	2.37	2.86	74.9	54	13,21,29		
36	I felt ignored by people	2.41	−0.07	0.73	1.67	2.55	190.2	150			
37	I felt guilty	2.39	0.07	0.86	1.76	2.54	160.8	139			
38	I had trouble keeping my mind on what I was doing	2.4	−0.47	0.45	1.7	2.7	187.6	152	26,32		
39	I felt that everything I did was an effort	2.3	−0.21	0.60	1.5	2.4	181.3	165			
40	My thinking was slower than usual	2.12	−0.22	0.78	2.09	3.11	155.6	139	46		
41	I felt slowed down	2.09	−0.48	0.38	1.47	2.55	*226.9*	168	43,44		
42	I felt like being alone	1.99	−0.83	−0.19	1.09	2.37	214.8	172			
43	I got tired more easily than usual	1.99	−0.56	0.24	1.37	2.39	216.3	182	41,44		
44	I felt that I had no energy	1.99	−0.81	0.15	1.20	2.39	192.4	182	41,43		
45	I had crying spells	1.99	0.83	1.49	2.34	3.33	122.0	109	34	*34.7*	4
46	I reacted slowly to things that were done or said	1.9	−0.18	0.93	2.3	3.3	160.8	143	40		
47	I was unable to do many of my usual activities	1.8	0.18	1.08	2.2	3.6	*193.7*	146			
48	I had little desire to eat	1.48	0.29	1.39	2.70	3.86	175.3	151			
49	I disliked the way my body looked	1.39	−1.07	−0.31	0.87	1.82	*299.4*	217		*76.4*	4
50	I ate more than usual	1.19	−0.54	0.70	2.33	3.73	*227.8*	180			
51	I lost weight without trying	0.57	1.90	4.24	7.03	9.39	151.8	124			

*Note.* Italicized ***S-Χ***^***2***^ and ***Χ***^***2***^ values are significant at *p* < 0.01 after Benjamini-Hochberg correction for multiplicity

*LD* Local dependence detected with indicated item numbers

The current calibrations are provided for didactic purposes and are not intended to replace the official PROMIS® parameters or to be used for research

We compared model fit statistics for two short forms and the original bank of 51 depression items. Model fit statistics quantify the discrepancy between observed and expected values for model-data fit. We used conventional fit criteria [42] including the model fit statistic, M_2_, [43, 44], Akaike information criterion (AIC) [45], Bayesian information criterion (BIC) [46], and root mean square error of approximation (RMSEA). A significant result for the model fit statistic (M_2_) indicates that the model does not fit the data (i.e., the null hypothesis is a correctly specified model). M_2_ can be sensitive if dimensionality is incorrectly specified [42]. AIC and BIC are appropriate when maximum likelihood estimation has been used (as in our example). In practice, BIC imposes a stronger penalty than AIC when models are complex. RMSEA adjusts for sample size. For AIC, BIC, and RMSEA lower numbers indicate better fit (significance tests are not possible but they do provide estimates of relative differences between solutions). See [42] for a description of pros and cons for goodness of fit statistics for IRT models. Stone [47] and Cai [48] also describe alternative approaches to assessing IRT model-data-fit.

In addition to model fit, a diagnostic statistic (*S-X*^*2*^) [49, 50] was used to examine item-level goodness of fit. *S-X*^*2*^ compares observed and expected response proportions for each item value. We used a significance value of 0.01 to correct for multiple tests [51]. Readers interested in a discussion of common approaches to the multiplicity problem and a discussion of alternatives are referred to [51, 52].

### Local independence

Local dependence (LD) indicates there is excessive covariance between two or more items. The LD statistic [53] examines bivariate fit to identify items that are excessively related after accounting for the latent trait. These diagnostic statistics are approximately standardized *Χ*^*2*^ values that become large (10 or higher) if a pair of items violates local independence. Borderline values between 5 and 10 may indicate local dependence or they may be a result of sparseness in one or more response categories. Consensus discussions among psychometricians and content experts are highly recommended to help determine content redundancy.

### Differential item functioning

Differential item functioning (DIF) is the detection of items performing differently in subgroups (e.g., men and women) when the latent trait level (depression) is held constant [54]. DIF is important to consider because it can be a threat to validity if members from subgroups respond differently to items (after controlling for the latent trait level). For instance, the presence of DIF for a depression item may indicate reduced validity for between-group comparisons, because responses may reflect individual-level characteristics rather than the depression trait that the PRO is intended to measure.

There are two types of DIF: uniform and non-uniform. Uniform DIF occurs when an item is consistently more likely to be endorsed by one subgroup across all levels of the latent trait. Uniform DIF is detected when one subgroup has higher difficulty/severity parameters (*b*_*ij*_ parameters) than another subgroup. In non-uniform DIF, the strength of the association (as measured by the *a* parameter [discrimination parameter]) between the item and the underlying trait differs by sub-group. In this paper, our example focuses on a didactic example of detecting DIF (uniform or non-uniform) for gender in depression items because there is evidence that women experience some depression symptoms differently than men [55, 56].

There are a variety of ways to assess DIF, such as Mantel-Haenszel, IRT-based methods, and ordinal logistic regression. For example, in several PROMIS® item banks, logistic ordinal regression (e.g., lordif in R [57]) was used. Scott and colleagues [58] and Crane and colleagues [59, 60] provide overviews of using logistic regression techniques to detect DIF in quality of life scales. There have also been developments in hybrid IRT/ordinal logistic regression models (e.g., [61]). After detecting DIF via statistical methods, we recommend consulting content experts to determine if there is a substantive justification for DIF or if it is more likely to be statistical noise.

In the current paper, DIF for gender was tested using Wald χ2 to evaluate statistical significance at the 0.01 level (see Fig. 2). We did not pre-select anchor items, and thus all items were used to estimate the depression mean and standard deviation separately for men and women. These estimates were then fixed, and all item parameters were estimated separately for men and women.

To control the type I error rate, we used an omnibus Wald χ^2^ statistic prior to examining individual items. For each item, an omnibus Wald χ2 statistic was computed to test for overall DIF. The supplemented EM algorithm was used to estimate more accurate standard errors to support the Wald test [62]. If the total χ2 was significant, the individual *a* and *b* parameter DIF statistics were computed using constrained tests. If significant DIF was detected based on *p* < 0.01 [63], an iterative process set the non-flagged items as the anchors and the Wald χ2 statistic was recomputed to correct for Type 1 error [64]. Following the iterative procedure, the magnitude of DIF was evaluated graphically by examining the item characteristic curves [65]. See papers by Thissen and colleagues [66] and Chen and colleagues [67] for discussions of alternative methods for controlling the type I error rate.

We used IRTPRO to plot ICCs for men and women on the same graph. We inspected the curves to see if they were similarly steep for women and men (indicating how related the item is to the latent trait) and whether the curves were in a similar place along the depression continuum. If *b*-DIF is present, we would expect to see one gender consistently endorsing higher levels of depression.

## Results

### Sample

The PROMIS Wave 1 cohort has been described elsewhere [12, 23]. See the introductory paper in this series for a demographics table [68]. A sample of 925 individuals completed the computerized PROMIS depression items and 11 demographic items. One-hundred individuals were deleted because they had a mean response time of less than 1 s or 10 or more items in a row where response time was less than 1 s. The final sample size was 825 individuals, one of whom did not report a gender. Gender was equally distributed (49% male, 51% female) and the mean age was 50.9 years (SD = 18.9).

### Missing data

IRT uses all available information, and thus listwise deletion is unnecessary. However, little missing data was noted. Less than five responses were missing for demographic items. Missing data for the depression items ranged from 40 respondents (5%) for the items “I felt worthless” and “I felt ignored by other people” to 46 respondents (6%) for the items “I felt guilty” and “I was unable to do many of my usual activities.” In concordance with missing data standards, multiple imputation was not used [69, 70].

Imputation estimates what a missing value might have been based on sources of information such as nonmissing observations in the dataset and demographic characteristics. See [69–71] for a discussion of handling missing data in PRO measures, including when and how to use multiple imputation. Specific to IRT models, Finch [71] found that multiple imputation produced the least bias for producing accurate estimates of item difficulty and discrimination parameters.

### Item misfit (51 original items)

Table 2 lists the *S-X*^*2*^ item misfit statistics. Out of 51 depression items, 9 items (18%) exhibited misfit after the Benjamini-Hochberg correction for multiplicity. Items exhibiting misfit were reviewed with content experts. They recommended setting aside the following 5 misfit items because they likely tap into constructs beyond depression: “ate more than usual,” “disliked the way my body looked,” “disappointed in myself,” “unable to do many of my usual activities,” and “felt slowed down.” One item was recommended to be set aside because it was likely to exhibit floor effects and the item also assumed recognition of depression symptoms from respondents: “I felt like I needed help for my depression.” Three items were judged by content experts to be integral pieces of depression, and thus were recommended to retain unless further psychometric problems were identified: “sad,” “depressed,” and “wished I were dead and away from it all.”

### Local independence (51 original items)

Four sets of items (14 items in total [27%]) were found to be locally dependent (Table 2), including fatigue, suicidal ideation, crying, and cognition. The subdomain with the most locally dependent items was suicidal ideation: “wished I were dead and away from it all,” “thought about suicide,” “no reason for living,” and “others would be better off if I were dead.” . For each set of locally dependent items, the best performing item was identified (usually the item with the best discrimination parameter) and then reviewed with content experts. Content experts recommended the following items to be retained from the LD sets: “I felt I had no reason for living,” “I reacted slowly to things that were done or said,” and “I had trouble making decisions.” For fatigue, content experts recommended keeping the item, “everything is an effort” over items assessing lack of energy because it was perceived to be more related to cognitive aspects of depression. No items assessing crying were retained because they also demonstrated DIF for gender (see below).

### Differential item functioning (51 original items)

Significant Wald *Χ*^*2*^ statistics for DIF by gender are presented in Table 2. When depression level was held constant, 3 items (6%) exhibited significant gender DIF. In all cases, the type of DIF detected was in the threshold (*b*) parameters (i.e., uniform DIF), indicating that women were more likely than men to endorse these depression items at all response levels. The items exhibiting DIF for gender included: “crying spells,” “felt like crying,” and “disliked the way my body looked.”

**Fig. 3:**
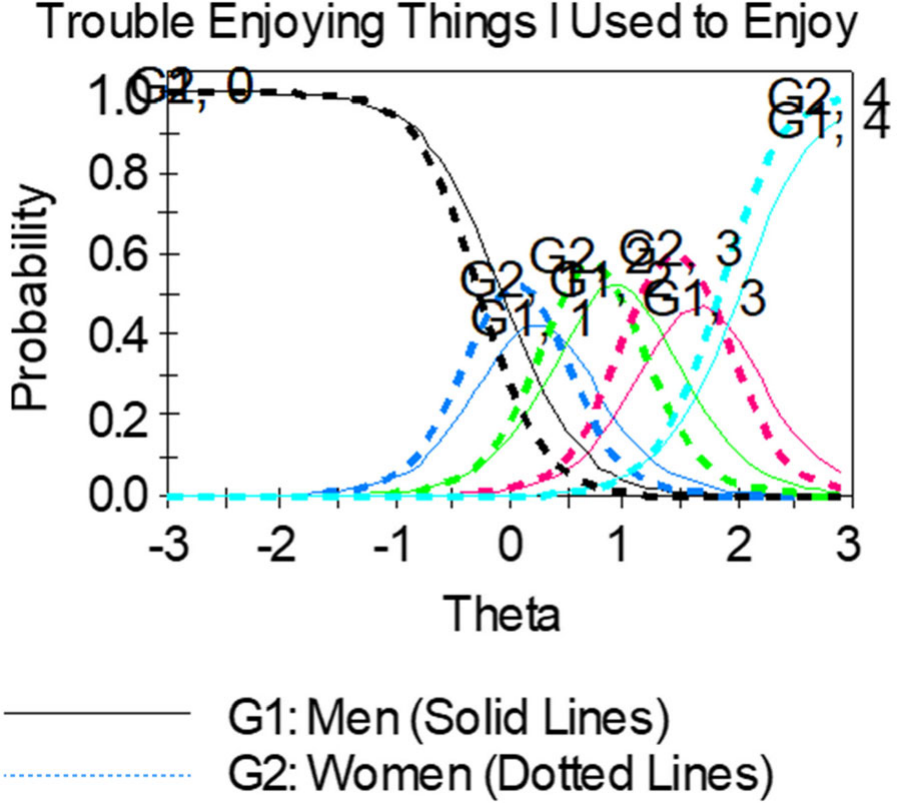
Example of an item showing uniform *b*-DIF. Women (dotted lines) consistently endorsed higher levels of depression (their curves are shifted to the left of men in every response category)

DIF was also examined graphically using item characteristic curves. Figure 3 shows an example of gender *b-*DIF. For this item, the item characteristic curves for each response category are similarly steep across gender, indicating that the items are equally discriminating, or related to depression, for both men and women. However, the item characteristic curves are shifted to the right for men, indicating that men needed a higher level of experienced depression to endorse response options than women.

Content experts perceived there was substantive justification for differential performance of crying items between men and women and recommended setting them aside. The general guidance is to remove items showing DIF from the item bank as long as content validity is not impacted. However, if the decision is made to retain an item exhibiting DIF for content considerations, then additional analysis steps should be taken to examine the impact on overall scores if DIF is ignored. See [72–74] for guidance on additional analysis steps. A less preferred option is to calculate different item parameters for men and women. As item banks like those from PROMIS are expected to evolve over time, the logistics for maintaining separate item parameters may become quite complex. This complexity should be weighed in the decision to use separate item calibrations for groups to control for DIF in the scores.

### Selecting items for short forms

Table 2 shows the 51 items in descending order from highest to lowest discriminating items (*a* parameter). To accommodate DSM-V criteria in short form 1, we made trade-offs between precision and content validity based on consensus discussions between psychometricians and content experts.

Table 3 shows the items selected for short forms 1 and 2. The most discriminating item in the bank of 51 items was “I felt hopeless,” which also met DSM-V criteria for symptoms and did not exhibit LD or DIF, and thus was included on both short forms. To meet DSM-V criteria for depressed mood, we looked at items with the best psychometric properties that assessed mood. The item “felt depressed” discriminated highly but exhibited item misfit, and thus we included “unhappy” on short form 1 and “depressed” on short form 2. The DSM-V mood criterion also includes diminished interest or pleasure in activities, and thus we included the item, “I had trouble enjoying the things I used to enjoy” on short form 1 and “I felt I had nothing to look forward to” on short form 2.

**Table 3:** Comparison of two potential short forms selected through a combination of psychometric properties and content validity considerations

DSM-V Criterion	Item	Short Form 1	Short Form 1 Rank Order *a*-parameter (based on 51 items)	Short Form 2	Short Form 2 Rank Order *a*-parameter (based on 51 items)
1. Depressed mood most of day	I felt unhappy	x	4	x	4
	I felt depressed			x	3
2. Little interest or pleasure in doing things	I had trouble enjoying the things I used to enjoy	x	18		
	I felt I had nothing to look forward to			x	9
3. Symptoms: psychomotor retardation or agitation	I reacted slowly to things that were done or said	x	46		
4. Symptoms: insomnia/ hypersomnia					
5. Symptoms: fatigue	I felt that everything I did was an effort	x	39		
6. Symptoms: worthlessness	I felt I was not as good as other people	x	24		
	I felt worthless			x	2
	I felt like a failure			x	6
7. Symptoms: excessive guilt	I felt guilty	x	37		
8. Symptoms: diminished ability to think/ concentrate or indecisiveness	I had trouble making decisions	x	26		
9. Symptoms: suicidal ideation	I felt I had no reason for living	x	13		
	I felt like I wanted to give up on everything			x	8
10. Symptoms: significant weight gain or loss/appetite loss	• I had little desire to eat • I ate more than usual • I lost weight without trying				
11. Significant distress or impairment	I felt that nothing could cheer me up			x	5
	I felt emotionally exhausted			x	11
Symptoms: hopeless	I felt hopeless	x	1	x	1
Symptoms: helpless	I felt helpless			x	7
Symptoms: withdrew from others	I withdrew from others	x	25		

Next we looked at well performing items that could meet DSM-V criteria for the symptoms category (insomnia/hypersomnia, psychomotor agitation or retardation, fatigue, worthlessness/excessive guilt, diminished ability to think/concentrate or indecisiveness, suicidal ideation, significant weight gain or loss/appetite loss). On short form 1, six items (out of ten) were chosen because they met DSM-V criteria even though they provided lower discrimination values than other items: “I reacted slowly to things that were done or said,” “I felt that everything I did was an effort,” “I felt I was not as good as other people,” “I felt guilty,” “I had trouble making decisions,” and “I withdrew from others.” The final DSM-V criterion is clinically significant distress or impairment in social, occupational, or other areas. On short form 2, two items were included: “I felt that nothing could cheer me up” and “I felt emotionally exhausted.”

Two DSM-V criteria could not be captured on short forms. Insomnia/hypersomnia could not be captured because a separate PROMIS® item bank was developed for sleep issues [75], and thus no items assessing sleep are included in the depression bank. The items assessing weight/appetite, “I had little desire to eat,” “I ate more than usual” and “I lost weight without trying” had the lowest discrimination parameters (and the item “I ate more than usual” was also significant for misfit). Content validity experts were concerned that the weight/appetite items were influenced by circumstances other than depression (e.g., health conditions, dieting, holidays). In addition, the item “I lost weight without trying” uses a severity response scale (“not at all,” “a little,” “somewhat,” “quite a bit,” “very much”) instead of a frequency scale like the other depression items, which may have contributed to misfit. Given the psychometric issues and content concerns, these items were not considered for short forms.

### Model fit

#### 51 items

Table 4 shows model fit statistics for all 51 PROMIS Depression items and two potential short forms. Overall model fit for the 51 items was relatively poor, indicating that local independence and DIF should be examined to determine if the item bank could be reduced to potentially improve model fit. Cronbach’s alpha, a measure of internal consistency/reliability from classical test theory, was above the ≥.90 criterion (marginal reliability = 0.98). Additional criteria such as the AIC, BIC, RMSEA, and M_2_ showed poor fit for the pool of 51 items. The AIC and BIC were both relatively high at 65,230.18 and 66,432.60, respectively (criterion: lower is better fit). The RMSEA was 0.43, which is much higher than the criterion of ≤.05 [48, 49]. Finally, M_2_ was significant at the *p* < .0001 level (163,378.86 [df = 1071] for 51 items, indicating poor model fit.

**Table 4:** Model fit changes for short form selection

Model	Cronbach’s alpha	AIC	BIC	-2log likelihood	Δ in -2log likelihood	RMSEA	M_2_ (df)
51 items	0.983	65,230.18	66,432.60	64,720.18	–	0.43	163,378.86 (1071)***
Short Form 1 Prioritizing Content (10 items)	0.946	13,762.99	14,234.40	13,562.99	51,157.19	0.01	1469.53 (1420)
Short Form 2 Prioritizing Precision (10 items)	0.945	13,825.12	14,296.54	13,625.12	51,095.06	0.01	1513.90 (1420)

Cronbach’s alpha = measure of internal consistency/reliability from Classical Test Theory (criterion: ≥.90).

*AIC* Akaike information criterion (criterion: the lower the number, the better the fit)

*BIC* Bayesian information criterion (criterion: the lower the number, the better the fit)

-2log likelihood = if models are nested, subtract at each step to see if step is significant

*RMSEA* Root mean square error of approximation (criterion: ≤ .05).

M_2_ = model fit.

***p < .001 (Note: a significant value for model fit indicates that the model does NOT fit well)

*df* Degrees of freedom.

#### Short forms

Short forms 1–2 performed similarly based on model fit statistics, but short form 2 provided more item precision (Table 4). In both short forms, the AIC, BIC, and RMSEA were low and the model fit statistic was not significant (indicating acceptable model fit). Internal consistency, as measured by Cronbach’s alpha, was high at 0.95 for both forms (and marginal reliability was high at 0.96). Nine out of 11 DSM-V diagnostic criteria were satisfied on short form 1 vs. five on short form 2.

**Fig. 4:**
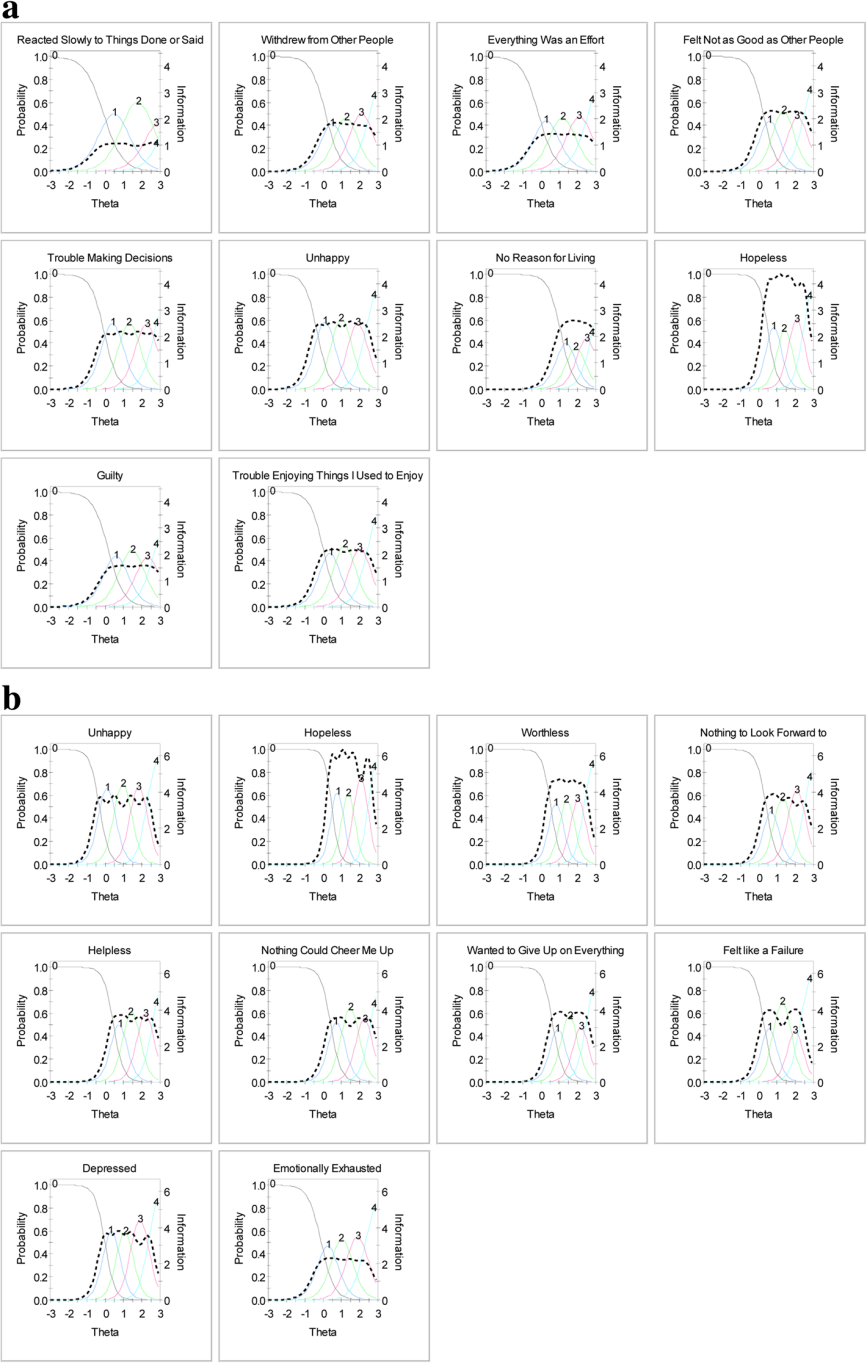
Item characteristic curves and information curves for items in **a** Short Form 1 **b** Short Form 2. Solid lines: item characteristic curves; dotted lines: information curves

Figure 4 displays item characteristic curves and item information curves for short forms 1 and 2. These plots graphically display the information conveyed in the item parameters. The location of the item characteristic curves and information curves confirms that these items measure the middle to upper end of the depression continuum.

In Fig. 4a for short form 1, the items “reacted slowly” and “everything was an effort” had information curves (dotted lines) around the .2 to .3 range, indicating less information was being obtained from these items than the other items in the higher range from .5 to .9. On short form 1, the items “no reason for living” and “not as good as other people” had trace lines bunched together and problems where “worse” response options did not have a higher probability of being selected as depression level increased (i.e., did not increase monotonically).

In Fig. 4b for short form 2, the item “felt emotionally exhausted” had a lower information curve than other items. The items “helpless,” “nothing could cheer me up,” “wanted to give up on everything,” and “felt like a failure” had trace lines bunched together and the probability for selecting response choices did not appear to increase monotonically as the level of depression increased.

**Fig. 5:**
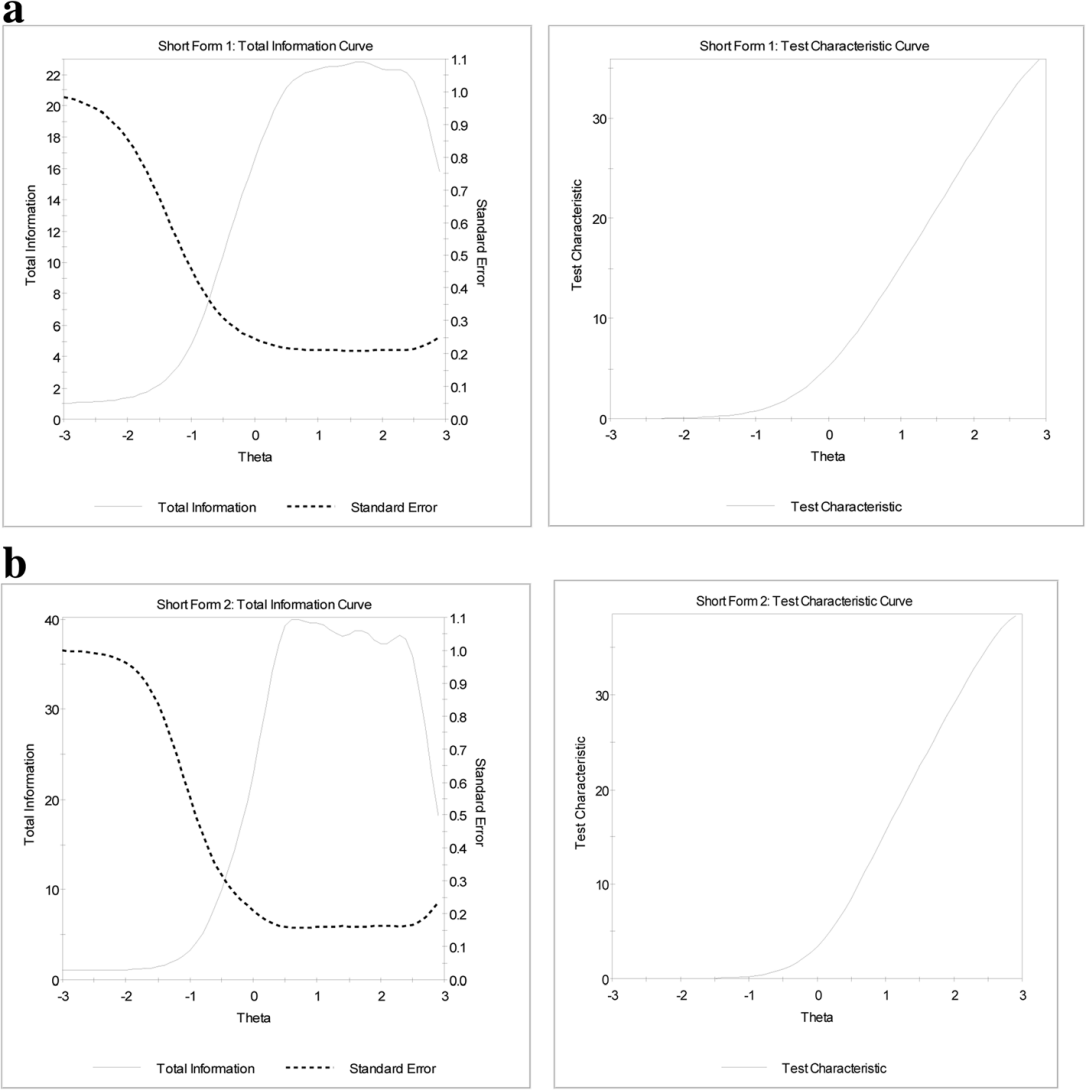
Comparing test information curves for short forms 1 and 2. **a** Short Form 1 **b** Short Form 2

Figure 5 displays the test information curves for short forms 1 and 2. A reliability level of 0.90 corresponds approximately to a value of 10 on the information scale, and thus the short forms have adequate reliability for theta values of − 0.5 and greater. In other words, the short forms measure depression well for people half a standard deviation below the mean depression score to the most depressed people at the upper end of the spectrum.

## Discussion

We applied IRT modeling to the measurement of depression as part of a series of papers comparing different psychometric methodologies for evaluating PRO measures (IRT, classical test theory, and Rasch modeling). IRTPRO software [22] was used to examine model fit, local independence, and DIF of 51 depression items developed by the PROMIS initiative [23].

Of the original 51 depression items, less than 30% exhibited problems such as local dependence (27%), DIF between women and men (6%), or item misfit (18%), which is consistent with the original PROMIS findings [23, 76]. In selecting items for short forms, items that exhibited DIF, local dependence, or misfit were removed. The result was a more stable construct and improved model fit. Since local dependence and DIF are both indicative of multidimensionality, this finding highlights the importance of dimensionality considerations when fitting IRT models to PRO measures. See the corresponding paper in this series that describes scale-level analyses for assessing dimensionality in the classical test theory framework [33], such as confirmatory factor analysis.

In cases of DIF, subgroup bias was detected in the threshold parameters (*b*) for 3 items (“I felt like crying,” “I had crying spells,” and “I disliked the way my body looked”). These items were removed from consideration for short forms in the current paper and the original developers also removed them from the official PROMIS® item bank containing the best performing 28 items [23].

In the current didactic exercise, two short forms consisting of 10 items were created using a combination of IRT methodology and DSM-V content considerations. Short form 1 prioritized content, and thus 6 items were included because they met DSM-V criteria but were in the middle or bottom half for discrimination parameters. Short form 2 prioritized high performing items and provided greater precision overall, but fewer DSM-V diagnostic criteria were satisfied. Short forms 1–2 performed equivalently in terms of model fit statistics but short form 2 items had more precision.

Two DSM-V criteria could not be captured on the short forms: weight/appetite and insomnia/hypersomnia The items assessing weight/appetite, “I had little desire to eat,” “I ate more than usual” and “I lost weight without trying,” had the lowest discrimination parameters and content experts flagged them to be set aside because circumstances outside depression affect them, like dieting or a health condition. In addition, the item “I lost weight without trying” uses a severity response scale (“not at all,” “a little,” “somewhat,” “quite a bit,” “very much”) instead of a frequency scale (“never,” “rarely,” “sometimes,” “often,” “always”) like the other depression items, which may have contributed to misfit. Future research should examine the degree to which differing response options influence model fit when developing and evaluating PRO measures.

We purposively chose a different content criterion (DSM-V [25]) than the original PROMIS® short form [23] that used affective and cognitive dimensions of depression. We broadened the target content validity criteria to mirror DSM-V criteria for depression, which meant considering content for cognitive and affective aspects of depression, somatic symptoms (psychomotor retardation), behavioral (social withdrawal), and suicidal ideation aspects of depression. Short form 1 emphasizing content captured 9/11 DSM-V criteria; short form 2 emphasizing item precision captured 5; and the earlier PROMIS short form [23] captured 4/11 criteria We present one way to combine psychometric data and content considerations to create short forms, but best practice recommendations would be helpful for the field.

Both an earlier short form from the PROMIS® group [23] and current short forms assess a depression range from the mean to about 3 standard deviations above the mean, indicating that “low” depression was not being captured well by the items in the short forms. “Low” depression may represent a different construct than moderate-high depression. For example, low depression may represent a “quasi-trait” or tap into personality characteristics, and thus multidimensionality may be introduced if it is included in item banks. It may also be that the items assessing “low” depression were tested in the current paper but had lower discrimination parameters and/or content validity concerns. Future research should better conceptualize what “low” depression is with people with lived experiences of depression. A better understanding of the entire continuum of depression may lead to interventions to prevent major depression and its negative impact on quality of life.

### Strengths and limitations of IRT in the development and evaluation of PRO measures

IRT models are highly flexible tools that provide item- and measure-level data, and thus can overcome shortcomings of classical test theory that produces scale-level data. By using item- and test-level information provided by IRT models, the most efficient administration approach can be used to reduce response burden with a high level of information/reliability. Both IRT and Rasch models allow short forms to be compared because they adjust for the difficulty of items. IRT models also allow for adjustment for discrimination parameters (because the *a*-parameter is allowed to vary in IRT but is held constant in Rasch models).

IRT also offers the ability to test item bias across demographic or clinical subgroups with one of several DIF methods [57–61]. Identifying DIF is important because the presence of DIF in a scale can reduce validity and reliability in measuring the latent trait [17]. DIF was a key component of PROMIS® methodology to select the best performing items for calibrated item banks [11, 12, 23]. For the PROMIS® Depression item bank, Pilkonis and colleagues [23] considered DIF evidence (due to age, race/ethnicity, and education) as part of a panel of psychometric analyses. In the current study, we narrowed it to DIF for gender to keep the didactic exercise manageable. Readers interested in a broader discussion of DIF and alternative uses (e.g., using DIF to develop separate scoring algorithms for subgroups exhibiting DIF) are referred to [54, 72–74].

Multiple software applications are available for IRT. Some choices for software applications include IRTPRO (see Fig. 2), PROC IRT in SAS, MPLUS, MULTILOG, PARSCALE, flexMIRT, and Xcalibre.

IRT models generally require a large sample size to obtain accurate and stable item parameters, which can be costly and time-consuming. As the number of response categories or the number of items increases, a larger sample size is needed due to the increase in the number of item parameters to be estimated. Items that have a modest relationship with the latent construct will also require a larger sample size since more information will be necessary to compensate.

The current study used a large sample, and thus the challenging issues of non-normal population distributions, substantial multidimensionality, longitudinal data, and small sample size are not addressed. Reise and colleagues [77] discuss alternative approaches to addressing non-normal distributions in IRT models. Issues of small size are tackled by Houts and colleagues [78] who describe the use of longitudinal IRT models as a pragmatic solution, and by Finch and French [79] who compare estimation methods for IRT models with small samples. See [80] for an overview of multidisciplinary IRT methods and [27] for a good discussion of a Bayesian multilevel multidimensional IRT model for locally dependent data. As the application of IRT to PRO measures continues to evolve, more research addressing these issues will be needed.

## Conclusions

Item response theory (IRT) is a family of flexible statistical models providing both item- and scale-level information, making it a powerful tool for PRO measure construction and refinement. Combining psychometric data with stakeholder input (including people with lived experiences of the health condition and clinicians) is highly recommended for scale development and evaluation. An example application and software output were described to encourage PRO researchers to consider IRT methods in the aim for accurate, precise scores that are sensitive to change and comparable across studies and populations.

## Data Availability

The datasets supporting the conclusions of this article are available from the PROMIS Health Organization, http://www.healthmeasures.net/explore-measurement-systems/promis. The calibrations and short forms presented in this series are not intended to replace official PROMIS® parameters.

## Abbreviations

AIC: Akaike information criterion
BIC: Bayesian information criterion
CAT: Computerized adaptive testing
DIF: Differential item functioning
DSM-V: Diagnostic and statistical manual version 5
EORTC: European Organisation for Research and Treatment of Cancer
GRM: Graded response model
ICC: Item characteristic curve
IRT: Item response theory
LD: Local dependence
NIH: National Institutes of Health
PRO: Patient-reported outcome
PROMIS: Patient-reported outcomes measurement information system
RMSEA: Root mean square error of approximation
